# Design and validation of a low-cost sugar-feeder for resource-poor insectaries

**DOI:** 10.12688/gatesopenres.16314.1

**Published:** 2025-03-03

**Authors:** Zachary Thomas Stavrou–Dowd, Clair Rose, Álvaro Acosta-Serrano, Lee Rafuse Haines

**Affiliations:** 1 Liverpool School of Tropical Medicine, Liverpool, L3 5QA, UK; 2 University of Notre Dame Eck Institute for Global Health, Notre Dame, Indiana, 46556, USA

**Keywords:** Attractive targeted sugar bait (ATSB), malaria, viable dyes, Allura Red, mosquito, Anopheles, vector control

## Abstract

**Background:**

The emergence of insecticide resistance in insects has led researchers to develop new control tools so that historic gains made in reducing disease transmission are not lost. Attractive targeted sugar baits (ATSBs) are a vector control tool being widely trialled to target insects that feed on plant sugars and blood. We designed a field-friendly, economical and more environmentally responsible sugar feeder for maintaining mosquito colonies and screening potential ATSB candidates.

**Methods:**

We simultaneously tested, in both male and female *Anopheles gambiae* mosquitoes, the effect of adding three water-soluble medical and food dyes (Allura Red, fluorescein and tartrazine) to the sugar solution to identify those insects that had ingested sugar from the feeder. To test feeder efficacy to deliver a toxic substance, we assessed the killing using boric acid, which kills both male and female mosquitoes when ingested. Using microscopy techniques compatible with fieldwork, including the use of a mobile phone camera, we documented the efficacy and tissue specificity of the dyes on mosquitoes after they were continuously fed dyed sugar solutions.

**Results:**

The easy-to-construct sugar feeder is an economical option for testing the efficacy of ATSB components on *Anopheles gambiae* mosquitoes *.* Allura Red AC was the preferred dye as it has low toxicity to mosquitoes and allows the researcher to quickly visualise the imbibed sugar meal within the abdomen. Feeding 1% fluorescein dye, but not 0.1%, for longer than five days induced systemic dye distribution, where the mosquito’s wing veins, antennae and legs brightly fluoresced when examined by a handheld black light torch (395-400nm emission).

**Discussion:**

Developing an affordable sugar feeder to maintain insectary-reared insects and test the efficacy of ATSB candidates involves designing a dye-labelled sugar bait station that is of low-toxicity, reusable and easy to construct using components available in low resource settings such as field stations.

## Introduction

Blood feeding arthropods can transmit pathogens to humans and animals, which make them of public health, agricultural and veterinary concern. Of these insects, some are exclusively obligate blood feeders (for example tsetse), whilst some are facultative blood feeders, feeding on both sugar and blood (i.e., mosquitoes and sand flies). The female malaria-transmitting mosquito *, Anopheles sp.*, survives on both plant nectars (sugars) and vertebrate blood. The female’s reliance on a high protein meal to lay eggs is what drives Anopheline mosquito species to seek blood, thus perpetuating the transmission of malaria in disease-endemic areas. Despite a 60% reduction in malaria mortality since 2000 (due to large-scale distribution of bed nets and other disease control interventions), this reduction has plateaued since 2017 primarily due to expanding resistance to insecticides. ^1^ Progress is further threatened by disturbances caused by the COVID-19 pandemic; the World Health Organisation states there was an increase in malaria deaths of 55,000 partly because of this disruption. ^2^

Transmission of malaria in most African countries is primarily driven by four Anopheline mosquito species: *Anopheles gambiae* s.s; *An. coluzzii; An. funestus* and *An. arabiensis.* ^3^ Whilst these dominant vectors are responsible for rural transmission, there is also an increasing risk of urban transmission as *An. stephensi* arrived in the Republic of Djibouti in 2012. ^4^ The two most implemented vector control tools, long-lasting insecticidal nets (LLINS) and indoor residual spraying (IRS), are routinely used to reduce mosquito populations. However, insecticide resistance continues to increase to a level where it now compromises the efficacy of mosquito-centric malaria control tools. ^5^^,^^6^ Consequently, there is renewed interest in the development and validation of alternative insect control tools such as spatial repellents and Attractive Targeted Sugar Baits (ATSBs). ^7^

Feeding on plant sugars is essential for male and female mosquitoes to survive, even though a female can supplement sugar feeding with blood-feeding. ^8^^,^^9^ Targeting an insect’s reliance on sugar has led to the development of a vector control tool called Attractive Targeted Sugar Baits (ATSBs). ATSBs are designed to combine sugar (nectar) with a range of toxicants (e.g. insecticides, biological or molecular agents, such as dinotefuran, *Bacillus thuringiensis* and double-stranded RNA ^10^^,^^11^) to deliver a lethal cocktail to the feeding insect. Epidemiological modelling has predicted that using ATSBs could reduce the prevalence of malaria by up to 30% in areas with high malaria burden. ^12^ An advantage of deploying ATSBs is that their value is not limited to malaria-transmitting mosquitoes - other insect vectors requiring sugar supplementation can also be targeted. Globally, several public and agricultural health-based ATSB trials have shown the efficacy of this type of intervention on several disease vectors; i.e. *Anopheles*, *Aedes* and *Culex* mosquitoes, as well as old-world sand flies ( *Phlebotomus papatasi)* and the biting midge, *Culicoides sonorensis.* ^10^^,^^13^^–^^16^

In standard mosquito ATSB assays where the uptake of mosquitocidal compounds by the insect is screened by combining the toxicant with a sugar solution, two methods predominate: a cotton ball soaked with a solution containing sugar and the active ingredient, or the use of filter paper placed in solution. ^13^^,^^17^^–^^20^ With increased global interest in ATSBs and a desire to reduce screening costs, we have designed and adapted ^21^ a small, low-cost and reusable sugar feeder for field and laboratory use to screen for compounds with activity against sugar-feeding disease vectors. We have also tested the efficacy of three dyes used in human medicine or food manufacture, to measure feeding efficiency and mosquito lethality when delivered in a 10% sucrose solution.

## Methods

### Mosquito rearing for bioassays

The insecticide-susceptible *An. gambiae* s.s. Kisumu strain (originally established in 1975 from wild stocks in Kenya) were reared from eggs provided by the Liverpool Insect Testing Establishment (LITE). The eggs were hatched in 500 ml of purified water with the addition of 2 ml of a 2% (w/v) baker’s yeast solution (Lesaffre, France). Following published methods, ^22^ we added 200 larvae to small trays (L 280 × W 175 × H 65 mm) with 500 ml of water and finely ground fish food (TetraMin tropical flakes, Blacksburg, VA, USA).

Upon larval pupation, pupae were added to cages (Bugdorm-4M3030, Watkins and Doncaster, Leominster, UK) until they emerged as adults and were maintained on 10% sucrose solution *ad libitum.* Adult mosquitoes were maintained at the Liverpool School of Tropical Medicine (LSTM) insectary at 26°C (± 2°C) and 80% relative humidity (± 10%), with an automated 12hr light-dark cycle that includes an hour of dawn and dusk. To induce egg-laying, cages of mosquitoes were allowed to mate for several days and then females were offered a bloodmeal using the Hemotek Membrane Feeding System (Hemotek Ltd., Blackburn, UK). Blood components were purchased from the National Blood Authority, which was constituted using a 1:1 ratio of human plasma (NC05) and research red blood cells (NC15) that were mixed upon arrival. The day following blood feeding, an oviposition cup was added to the cage to collect eggs.

### Manufacture of sugar feeders

**Figure 1.**
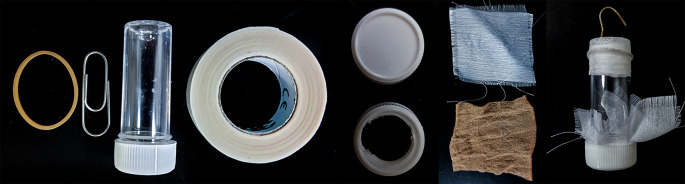
Components used to construct small sugar feeders for use in resource-limited insectaries. From left to right: elastic band, paperclip, 8 mL bijou, medical tape, bijou lid with drilled hole, various materials, assembled sugar feeder *.*

Sugar feeders were constructed using an adapted method comprised of five components: a paperclip (for hanging the feeder), tape (to attach a paperclip to the feeder), a 5 ml universal tube (to contain sugar solution), fabric or nylon (to cover the opening of the tube) and an elastic band (to hold the fabric to the feeder) ( Figure 1). A sugar feeder can also be constructed using a vial with a lid by drilling a wide hole through the lid; this replaces the elastic band requirement. A full protocol, including images, video of construction and costings can be found at dx.doi.org/10.17504/protocols.io.14egnzrbmg5d/v1.

### Dye assessment

To confirm whether mosquitoes had ingested sugar from the feeder, we trialled the use of 10% sucrose with the addition of one of three dyes; **red**: Allura Red AC (Merck Life Science UK, Gillingham UK), **green-yellow**: fluorescein sodium salt (Honeywell, Charlotte, NC, USA); and **yellow**: tartrazine (Acid yellow 23, Alfa Aesar, Ward Hill, MA, USA). The individual dyes were tested at concentrations between 0.1% (50 mg in 50 ml of 10% sucrose) and 2% (1g in 50 ml of 10% sucrose). Groups of 35 non-blood fed, 2-5 day old female *Anopheles gambiae* were added to large cages (Bugdorm-4M1515, Watkins and Doncaster, Leominster, UK) with one of our bespoke sugar feeders containing 5 ml of a 10% (10 g in 100 ml of water) sucrose/dye solution hung inside each cage so mosquitoes could feed *ad libitum.* Mortality was measured daily over seven days.

### Boric acid mortality

We assessed the ability of the sugar feeder to deliver a toxicant by using a known insecticidal compound, boric acid, at the standard field concentration of 1%. ^23^^,^^24^ Groups of 35 non-blood fed, 2-5 day old, females were added to cages (Bugdorm-4M1515, Watkins and Doncaster, Leominster, UK) and sugar starved for 12 hours. Post starvation period, a 10% sucrose solution containing 1% (0.5 g in 50 ml) boric acid and 1% (1 g in 100 ml of sucrose) Allura red AC (Merck Life Science UK, Gillingham UK) was added to each cage and mosquitoes were allowed to feed *ad libitum.* The control cage was fed 10% sucrose solution containing only 1% Allura red. Mosquito mortality was recorded at 24, 48 and 72 hours.

### Detection of dye ingestion using fluorescent and light microscopy

To assess whether mosquitoes fed from the feeders, mosquitoes with coloured abdomens (detected by eye) were removed and chilled at -20°C for 10 min. Upon cold knockdown, whole insects were viewed using a stereoscopic dissection scope equipped with an epi-fluorescence filter (ultraZOOM-3 Research Grade Stereo Microscope with FluDual 3W Fluorescence Epi-Illuminator (SKU UZ-3-Dual LED ST-1), GX Microscopes). All images were either captured using a Nikon JF SLR camera mounted to the microscope or mobile phone camera. The software HeliconFocus 8.1.1 was used to stack the images into a single photo with a greater depth of field. Adobe Photoshop CS6 (vs 13.0) was used for post-acquisition image processing, which included manipulating contrast, colour balance and noise reduction.

### Correction of mortality using Abbot’s Correction

When interpreting the results of the assays, we have used a control threshold of 20% mortality as this is the established threshold for the negative control using Abbott’s Correction. ^25^ Any mortality above this threshold was deemed to have caused failure of the control (if used in a standard assay) and therefore caused excess toxicity to the mosquito. Statistics and graphs were constructed using GraphPad Prism (Version 9.4.1).

## Results

### Toxicity of viable dyes Allura Red AC, tartrazine and fluorescein to mosquitoes

**Figure 2.**
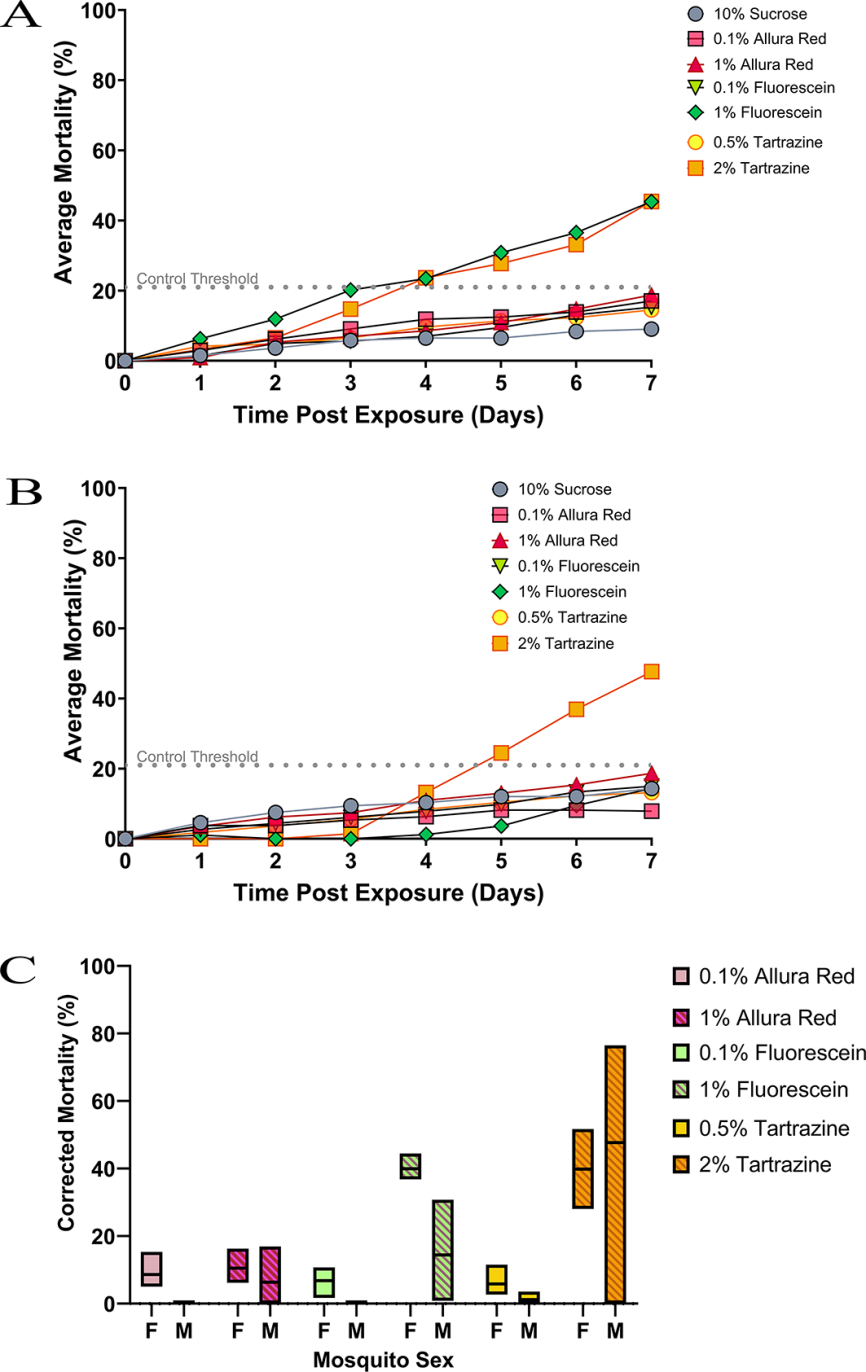
Mosquito mortality when fed exclusively on dye-infused sucrose. Sugar feeders containing different dyes and concentrations were tested against female (A) and male (B) *An. gambiae* mosquitoes. Mortality was assessed (y-axis) daily over a week (x-axis) of continual feeding. The dotted line represents the 20% mortality threshold that shows which dyes are more toxic over time. Direct comparison of final mortality at day 7 between male and female mosquitoes according to the dyes tested (C).

After 24 hours of feeding on the different dyed sugar solutions ( Figure 2A), all dye concentrations tested produced higher mortality in female mosquitoes: 10% sucrose control (1.5%), 0.1% Allura red (2.9%), 1% Allura red (0.9%), 0.5% tartrazine (4.2%), 2% tartrazine (3.2%), 0.1% fluorescein (1.4%) and 1% fluorescein (6.3%). However, on the first day, differences between the control mortality and dye mortality were not significant either with dye or concentration. During the subsequent week of continual feeding, all dye concentrations caused increased mosquito mortality. The difference in mortality between the control and dyes was significant only when fed either 1% fluorescein (p = 0.0038) or 2% tartrazine (p = 0.0311), which became apparent at day three for 1% fluorescein and day four for 2% tartrazine.

Male mosquitoes ( Figure 2B) showed a similar trend to females, with minimal differences between control and dye concentrations observed at 24h. As with female mosquitoes, male mortality increased with all dyes tested, but only the highest concentrations of fluorescein (1%) and tartrazine (2%) gave significantly higher mortality than the threshold: sucrose control (10.5%), 0.1 % Allura red (4.8%), 1% Allura red (14.6%), 0.5% tartrazine (8.3%), 2% tartrazine (51.2%), 0.1% fluorescein (8.2%) and 1% fluorescein (23.3%). The breach in the control threshold occurred on day seven for 1% fluorescein and day five for 2% tartrazine.

To assess if mortality differences between male and female mosquitoes with each dye was statistically significant, the average percentage mortality of each dye concentration was corrected from its control using Abbott’s Formula. This correction was used as male and female experiments were run separately with complementary controls. The corrected mortality examined at 24 hours and seven days post-exposure ( Figure 2C) showed no significant differences between male and female mosquitoes at any dye concentration. Even though 1% fluorescein was more toxic in females than males, when mortality was corrected for controls, there was no significant difference between the two on any date post-exposure.

### Delivery of toxic dose of boric acid

**Figure 3.**
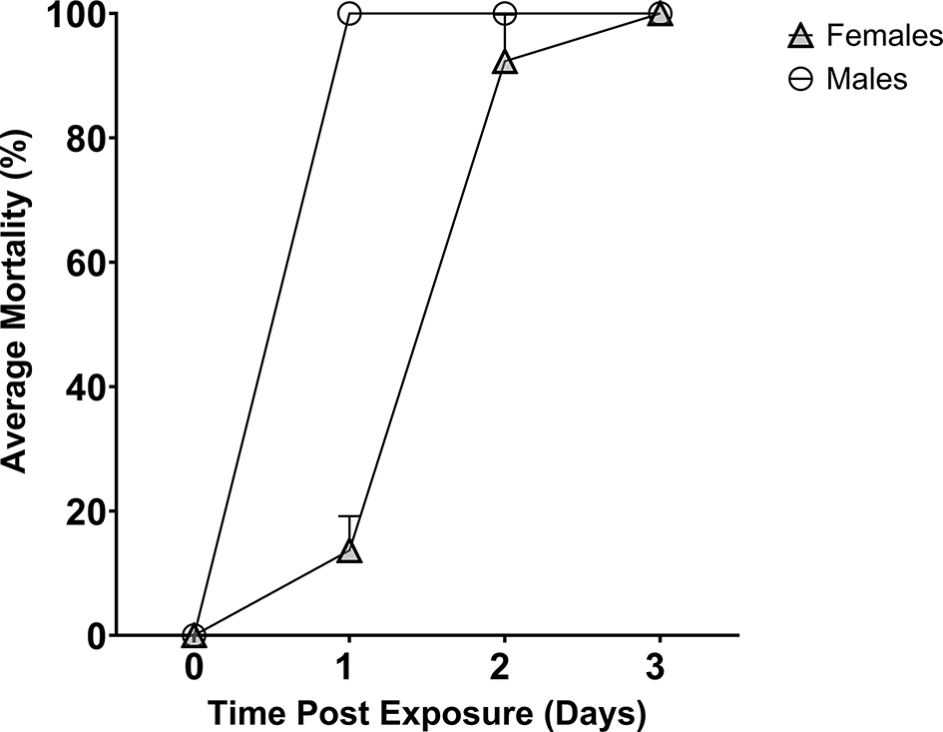
Mosquito mortality after feeding on 1% boric acid delivered via the bespoke sugar feeder. Boric acid is toxic to male (circle) and female (triangle) mosquitoes over a time period of three days.

Boric acid (1% w/v in 10% sucrose) was the toxicant used in the sugar feeders to test its ability to deliver a toxic compound ( Figure 3). In three replicates, all male mosquitoes died within 24 hours. Female mosquitoes were slower to die with an average mortality of 13.7% at 24 hours (CI _95_ 0.00 – 27.4%), 92. 3% at 48 hours (CI _95_ 73.7 – 92.3%) and 100% by 72 hours. The difference in mortality between male and female mosquitoes was significant at both 24 hours (p <0.0001) and 48 hours (p = 0.0453), indicating male mosquitoes die quicker upon ingesting boric acid-containing sucrose.

### Confirmation of mosquito feeding from feeders

**Figure 4.**
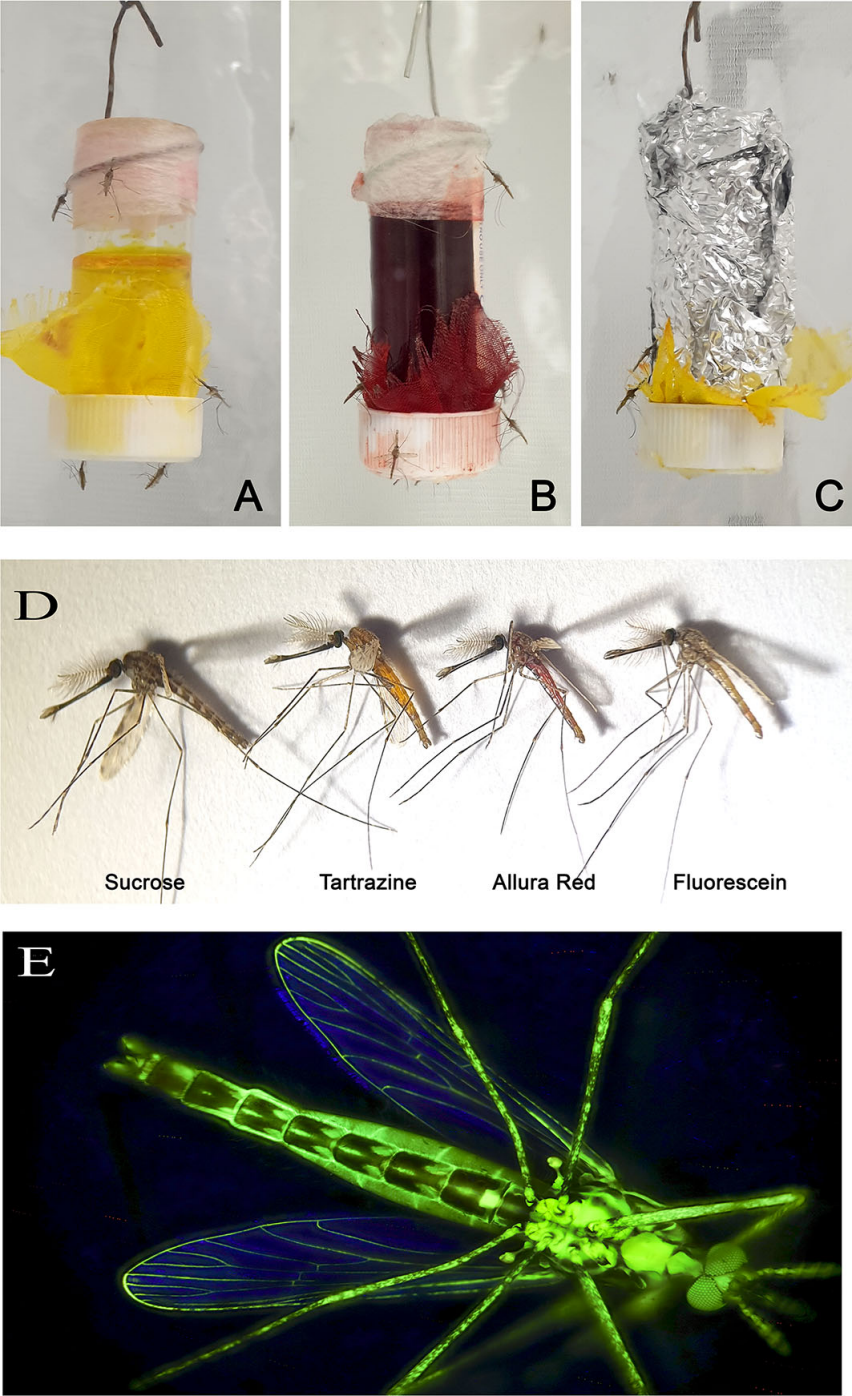
The sugar feeders (A-C), abdominal colouring (D) and fluorescein-stained mosquito. Mosquitoes feeding from sugar feeders containing the dyes tartrazine (A), Allura red (B) and fluorescein (C). After ingesting a sugar meal, the dyes colour the mosquito abdomen strong enough to be detected by eye (D). When viewed under UV fluorescence or a blacklight torch, fluorescein is systemically distributed through the female mosquito (E).

After 24 h of adding the sugar feeders to the mosquito cages, the mosquito abdomens were distended and the colours of each dye solution was clearly visible by eye ( Figure 4A-D). Feeding was also evidenced by the deposition of strongly visible coloured excreta along the edges of the cage floor and directly underneath the sugar feeder. This was especially evident when mosquitoes fed on 1%, 2% and 1%, of Allura red, tartrazine and fluorescein, respectively. Furthermore, the excreta from fluorescein-fed mosquitoes fluoresced when a black light was shone on the cage floor, which made tracking resting behaviour possible. When mosquitoes were examined using a dissecting microscope (40-200X), the extent of dye dispersal through the mosquito changed according to how long the mosquitoes had fed on the dye-infused sucrose.

**Figure 5.**
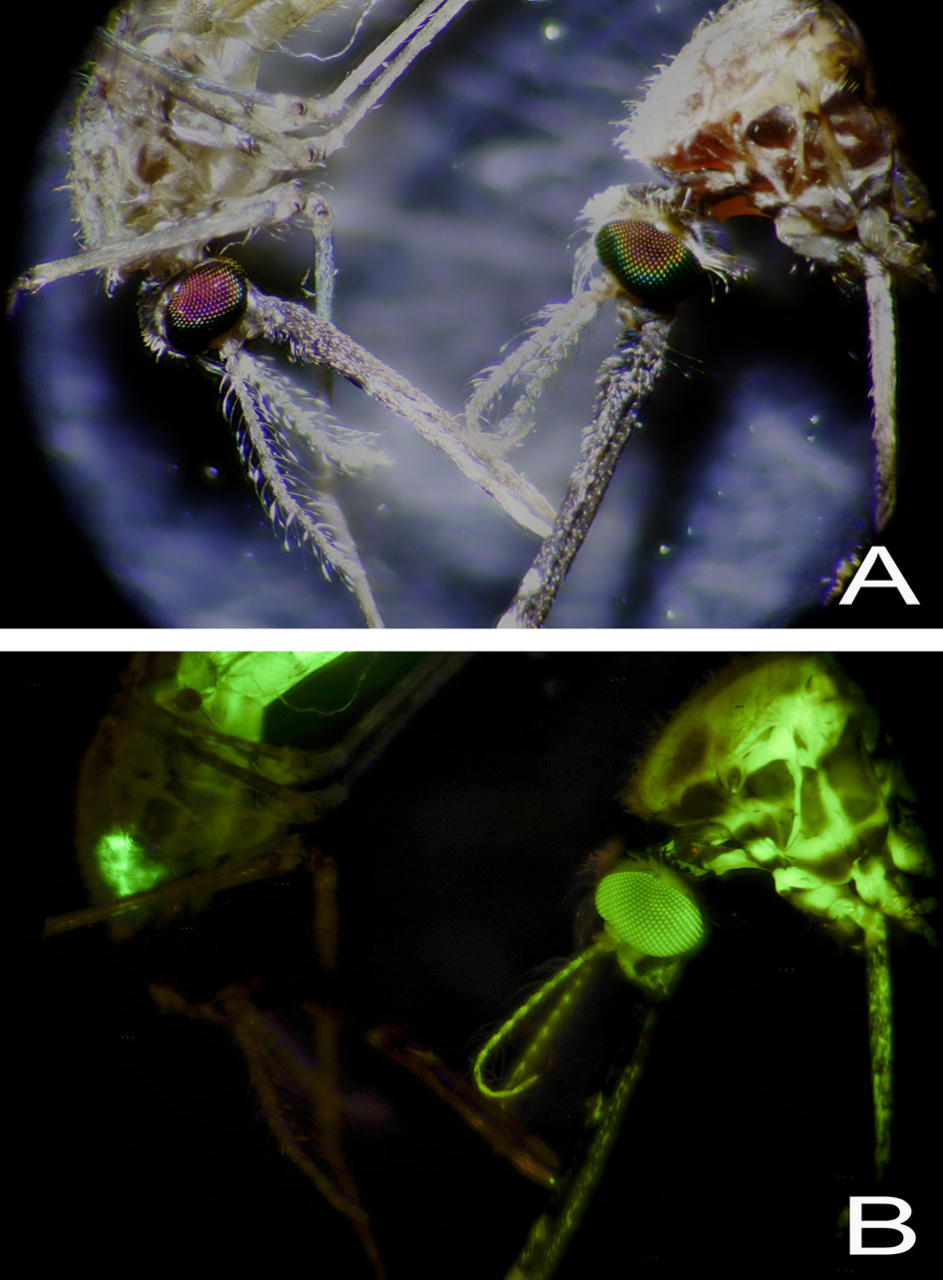
Female *Anopheles gambiae* fed on fluorescein-containing sucrose. Brightfield microscopy of two female mosquitoes (10X) (Panel A) and UV illumination (Panel B). The mosquito on the left was fed 0.1% fluorescein for 5 days and the specimen on the right received 1% fluorescein for the same amount of time.

After feeding for 3-5 days on the sugar feeders, the interior of the mosquito legs also took on the hue of the fed dye, which indicates the dye had leached from the crop/gut into the haemolymph. This was confirmed by examining mosquitoes fed 1% fluorescein dye ( Figure 4E). When these mosquitoes, both male and female, were exposed to UV light (or a handheld blacklight torch), the entire mosquito fluoresced, including wing veins, legs, antennae and eyes. This systemic labelling was not seen when mosquitoes were offered a lower concentration (0.1%) fluorescein for five days ( Figure 5).

## Discussion

Here we describe the design and testing of a simple sugar feeder that can be used to deliver dyes or lethal compounds (solubilised in 10% sucrose solution) to male and female *An. gambiae.* The feeder can also be implemented as an economical alternative to provide sugar for mosquito rearing. We have demonstrated there is no increase in mortality using the feeder when compared to the standard cotton pad method soaked in sucrose (data not shown). An added benefit of using hanging feeders is that they curb the establishment of microbial (fungal) overgrowth that is routinely seen on sugar pads and the cage roof after a few days, which means the overall health of the insectary can be better maintained.

We have also demonstrated how the sugar feeder does not impede the delivery of the toxicant boric acid as all mosquitoes feeding on this solution were killed within three days. Why males died a day earlier than females could be indicative of an innate sex-specific susceptibility or evidence of sex-specific sugar-feeding behaviours. We have identified three demonstrable benefits of using this sugar feeder for testing toxic sugar solutions, particularly ATSB compounds:

1. The sugar feeder requires only five, readily available components, which reduces the overall cost to the laboratory ( dx.doi.org/10.17504/protocols.io.14egnzrbmg5d/v1) The cost can be further offset as the feeder is fully reusable once washed using standard laboratory cleaning reagents such as bleach. For research groups adopting environmentally conscious methodologies, this would be relevant.
2. The feeder supplies a constant dose without causing wicking of the dye or toxicant, which could either lead to the separation of toxic sugar components or concentration effects due to evaporation - both may occur with standard cotton wool or filter paper feeding techniques.
3. The volume required to fill the feeder is low (<10 ml), which reduces the initial amount of toxic solution required, yet still lasts for a week in a cage of 100 mosquitoes under standard humidity and temperature for rearing. This may be particularly useful when mosquitocidal compounds are expensive to test or difficult to acquire. Furthermore, it is easy to scale up the design by using 50 ml tubes for feeding extending beyond one week.

To confirm that mosquitoes were readily ingesting the sugar provided in the feeders, we tested three viable dyes diluted in a 10% sucrose solution: Allura Red AC, fluorescein sodium salt and tartrazine. Each dye produced differing mortalities; both concentrations of Allura Red AC were well-tolerated, as well as the lower concentrations of tartrazine (0.5%) and fluorescein (0.1%). For either male or female mosquitoes, the feeder, when filled with 5 ml of solution, could be used for up to seven days of continuous feeding without causing >20% mortality. However, it is still important to assess mortality (ie. toxicant efficacy) in conjunction with the ability to detect the ingested solution and thereby resolve if the mosquito has died of starvation or injury, which can be seen by the absence of a dyed carcass.

For ATSB trials lasting several days, based on our assessment, Allura red is the best dye. It causes the lowest mortality, provides an easy method to identify sugar-fed mosquitoes by eye without the need for specialised equipment (as they have noticeably red abdomens and haemolymph) and rapidly confirms sugar feeding as it is evidenced, after only 24 h, by dark red excreta collecting within the cage. Previous research has also indicated red dyes, such as Allura red, do not themselves cause optical attraction to sugar baits and therefore are suitable as a control where bait attraction is being measured. ^26^^,^^27^

With the tartrazine, identification of mosquitoes by eye was more difficult because the ingested yellow dye does not contrast as well against *Anopheles* mosquito abdomens, although this can be easily overcome by using microscopy. With the higher mortality compared to other dyes, a 2% tartrazine solution would be unsuitable for detecting sugar feeding in mosquitoes beyond a few days. Using a lower dose, however, could be used as the mortality in males and females was less than 20% at 0.5% tartrazine. In field trials, tartrazine (sprayed on plants at 0.4% in one study and 2% in a 20% sucrose solution in another) had been suitable for identifying single exposure where other sugar sources are available without compromising control mortality. ^28^^,^^29^

Fluorescein dye has been previously used to mark sugar meal ingestion in culicine mosquitoes, which is why we specifically trialled this dye with Anophelines. ^30^ The authors demonstrated when mosquitoes fed on a 0.01% fluorescein diet, they did not suffer from dye-related toxicity or loss of fecundity. We further explored fluorescein toxicity by increasing the fluorescein concentrations tested by 10- and 100-fold in our experiment to establish whether we could 1) visualise the dye by eye in mosquito abdomens, and 2) determine if at higher concentrations this dye would become toxic. When mosquitoes were fed for 3-5 days on a 1% fluorescein sugar diet, the dye moved systemically out of the crop and midgut into the circulatory system as evidenced by mosquito eye, wing, antenna and palp fluorescence. This did not occur when mosquitoes were maintained on 0.1% fluorescein. Although we did not specifically measure the loss of fluorescence over time, we did observe that five days after discontinuing fluorescein-labelled sugar feeding, the mosquitoes remained fluorescent by eye when exposed to a blacklight torch. This warrants further investigation as the longevity of dye labelling could be advantageous for short-term marking insects in the field, keeping in mind limitations possibly associated with exposure to sunlight and other environmental conditions. We, however, reported that continuously feeding 1% fluorescein increased mosquito mortality and therefore it would not be suitable at this concentration for long-term ATSB assays where such additional mortality could confound the efficacy of the bait being tested.

In conclusion, with the renewed attention on testing compounds for use in ATSBs, we have designed and tested a small, low-cost, easily assembled sugar feeder that can either be used for general insectary maintenance or to deliver a toxic compound during sugar feeding. This sugar feeder allows insectary staff with limited resources (or entomologists working in the field to test toxic compounds) to use commonly found, inexpensive components. Such feeders may contribute to the collection of more robust field data as it has been reported that feeding mosquitoes with cotton wool pads can lead to low feeding rates. ^20^ In addition, where the filter paper method has been used to administer toxic sugar solutions through osmotic pressure, ^19^^,^^31^ there is potential for chromatographic separation of the solution compounds (especially dyes), which could distort the active ingredient concentrations ingested by mosquitoes.

We demonstrate that 1% Allura Red AC added to 10% sucrose is an easy way to confirm ingestion by either male or female *An. gambiae*, particularly where sugar-feeding experiments continue beyond a few days. As Allura Red AC is an azo dye, it also shows stronger emission intensity as the solution viscosity increases, ^32^ which makes it robust within the context of insect digestive processes. Furthermore, Allura Red AC also exhibits fluorescence (543 nm (excitation) and 593 nm (emission)), so the efficacy of sugar feeding can be more accurately determined using fluorometry, ^27^ and may potentially be used to quantify the volume of sugar ingested in future studies investigating whether an ATSB intervention influences mosquito feeding behaviours.

## Authorship contribution

Conceptualization: ZSD, CR, LRH

Formal analysis: ZSD, LRH, CR

Investigation: ZSD, LRH, CR

Methodology: ZSD, LRH, CR

Visualization: ZSD, LRH

Writing - original draft: ZSD, LRH

Writing - review & editing: ZSD, LRH, AAS

Resources: AAS

Funding acquisition: AAS, ZSD

Project administration: LRH, AAS

Supervision: LRH

## Data Availability

Figshare: Mortality of male and female *Anopheles gambiae* fed sucrose solution with dye using a low-cost sugar-feeder for resource-poor insectaries, https://doi.org/10.6084/m9.figshare.25575297. ^33^ Figshare: Mortality of male and female *Anopheles gambiae* fed sucrose solution of boric acid using a low-cost sugar-feeder for resource-poor insectaries, https://doi.org/10.6084/m9.figshare.17889614. ^34^ Data are available under the terms of the Creative Commons Attribution 4.0 International license (CC-BY 4.0).
